# Vertebral refracture after percutaneous vertebroplasty for osteoporotic vertebral compression fractures with and without brace wearing: A retrospective study of 300 patients

**DOI:** 10.3389/fsurg.2022.1056729

**Published:** 2023-01-10

**Authors:** Guo Xinyu, Zhu Na, Zhang Haihong, Hao Dingjun

**Affiliations:** ^1^ Department of Spine Surgery, Honghui Hospital, Xi’an Jiaotong University, Xi’an, China; ^2^ Shaanxi Key Laboratory of Spine Bionic Treatment, Xi’an Jiaotong University, Xi’an, China; ^3^ Department of Orthopedics, The Second Hospital of Lanzhou University, Lanzhou, China; ^4^ Department of Imaging, Xianyang Center Hospital, Xianyang, China

**Keywords:** osteoporotic vertebral compression fractures, percutaneous vertebroplasty, brace, osteoporosis, prognosis

## Abstract

**Background:**

The aim of the study was to examine the clinical incidence rate of vertebral body fractures after percutaneous vertebroplasty (PVP) with and without brace wearing and provide a new guiding ideology for preventing vertebral fractures after clinical surgeries.

**Methods:**

The retrospective analysis included 100 outpatients who underwent PVP between January 2017 and December 2018 without bracing after PVP surgeries (non-brace-wearing group). In total, 100 patients were paired into the rigid brace group and 100 patients were paired into the soft braces group according to propensity score matching. Seven independent variables were used in the soft and rigid brace group: age, sex, body mass index (BMI), visual analog scale (VAS), Oswestry Disability Index (ODI), and Cobb angle. The VAS, ODI, and Japanese Orthopaedic Association (JOA) scores were recorded preoperatively on the second day, after 1 month, after 3 months, and during the last follow-up postoperatively. We recorded the incidence of vertebral refracture in each of the three groups of patients and evaluated the effect of braces on postoperative fractures based on the ODI, VAS, and JOA scores.

**Results:**

All patients were followed up for 8–24 months (mean 22.4 months). Compared with the preoperative values, the age, sex, BMI, bone density, ODI, VAS, and Cobb angle between refracture and non-refracture were not statistically significant. The VAS, ODI, and JOA scores significantly increased in the brace-wearing group compared with those of the non-brace-wearing group (*p* < 0.05). The incidence of vertebral refracture in the brace-wearing group was lower than that in the non-brace-wearing group, between which there was a significant difference (*p* < 0.05). Three months postoperatively, the JOA score of the soft brace group was significantly higher than that of the rigid brace group (*p* < 0.05). During the last follow-up, it was found that there was no difference in the VAS score, the incidence of refracture, or ODI between the soft brace group and the rigid brace group (*p* > 0.05). The improvement in the JOA score of the soft brace group was better than that of the rigid brace group, between which there was a significant difference (*p* < 0.05).

**Conclusion:**

Braces can improve the prognosis of quality of life and postoperative subjective perception, whose presence can relieve postoperative residual pains. In contrast, patients can have a better medical experience wearing a soft brace.

## Introduction

The risk of fractures increases with osteoporosis, a systemic bone disease characterized by loss of bone mass and microstructural changes in the bone trabeculae (1). Approximately 300 million aged over 65 years and middle-aged people around the world experience osteoporosis, according to relevant literature reports (2), while it is more prominent in China. Combined with the sixth population census in China (3), it is estimated that the incidence of osteoporosis among people aged over 40 years on mainland China is 19.74% (approximately 20%), that is, nearly 112 million people. In the country, fractures caused by osteoporosis account for 20% of all types of fractures (4). Mild bending or coughing can lead to fractures for patients with osteoporosis, the vast majority of which are spinal fractures (5). Osteoporotic vertebral compression fractures (OVCFs) cause great harm to the physical and mental health of patients who have experienced these complications (6, 7). Although the clinical effect of percutaneous vertebroplasty (PVP) is significant, the incidence of vertebral refracture after surgeries is still in the range of 0%–63%. There is no clinical study in which compliance and materials of brace wear after the PVP of OVCFs or its relationship with vertebral refracture of patients with osteoporosis are analyzed. Therefore, it is necessary to study the effect of brace wearing on vertebral refracture after the PVP of OVCFs focused on individual evaluations after surgeries, which has a positive significance for the prevention of vertebral refracture.

## Materials and methods

### Inclusion and exclusion criteria

The inclusion criteria were as follows: (1) with or without the history of sprain, fall, and other minor traumas; (2) the spine was painful with or without restricted motions of the waist or back in the past 1–2 weeks; (3) the anterior and lateral x-ray films of the spine showed a vertebral compression and wedge-shaped changes—magnetic resonance imaging (MRI) showed that the vertebral fractures were fresh, with a low signal intensity on T1, an isointense or high signal intensity on T2, and a high signal intensity on a fat-suppressing image; (4) bone mineral density *T* ≤ −2.5; (5) the follow-up period was more than 6 months; (6) all patients were standardized to take anti-osteoporosis drugs including calcium carbonate D3, calcitriol, and alendronate during the follow-up period; (7) treatment with PVP *via* unilateral portals; and (8) the braces worn by the patients were strictly according to the requirements from the first postoperative day, and brace wearing continued for no less than 3 months.

The exclusion criteria were as follows: (1) patients who did not meet the inclusion criteria; (2) the follow-up period showed patients experiencing a fracture caused by a high-energy injury; (3) the MRI examination showed that the vertebral fracture was an old fracture; (4) computerized tomography (CT) and MRI showed that the fractures affected the posterior column of the vertebral body or that the fracture blocks compressed the spinal canal; (5) the spinal cord and nerves were injured; (6) case fractures caused by tumor metastasis or infection; (7) patients with multiple fractures or obvious medical diseases who could not tolerate surgeries; (8) during the follow-up period, some patients were lost to follow-up due to various factors; (9) they did not take enough anti-osteoporosis drugs regularly after discharge; and (10) patients who took steroids.

The following criteria determined a vertebral refracture: (1) after PVP, without an obvious history of traumas, with localized low back pain, with or without limitations of low back movement; and (2) the x-ray or CT examination showed that the height of the anterior and posterior edge of the original or other segments of the vertebral body were changed, and MRI showed signal changes of fresh fractures.

A total of 1,167 outpatients with OVCFs underwent a PVP between January 2017 and December 2018, among whom 100 patients did not wear braces after the PVP (non-brace-wearing group). The inclusion and exclusion criteria were met by 728 patients who wore braces among all the outpatients. Age, sex, body mass index (BMI), bone mineral density (BMD), visual analog scale (VAS), and Oswestry Disability Index (ODI) were set as independent variables for propensity matching; 100 patients were assigned to the rigid brace group and 100 to the soft brace group. According to the regulations, all patients in the three categories were subject to strict regulations.

X-rays were taken for the patients undergoing a PVP in the supine position, which revealed fractures. Infiltration anesthesia was performed with 2% lidocaine under x-ray fluoroscopy to reach the anterior edge of the vertebral body, and an injection into the fractured vertebral body was performed during the drawing period of bone cement. X-ray fluoroscopy showed that the recovery of vertebral height and the distribution of bone cement were satisfactory. Finally, a needle was removed from the puncture wounds, which were dressed, and the patients were placed in the supine position back in the hospital.

**Figure 1:**
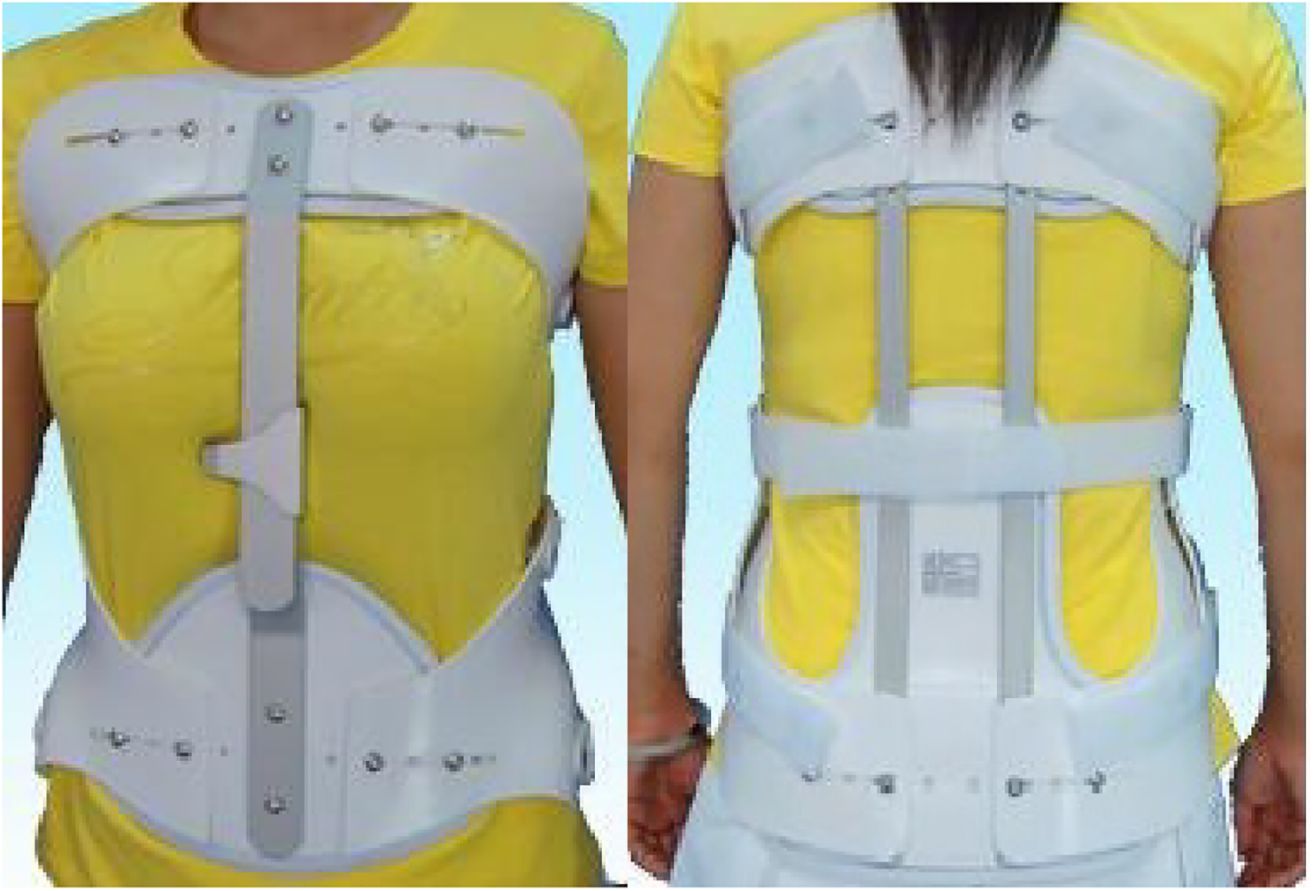
Rigid brace.

**Figure 2:**
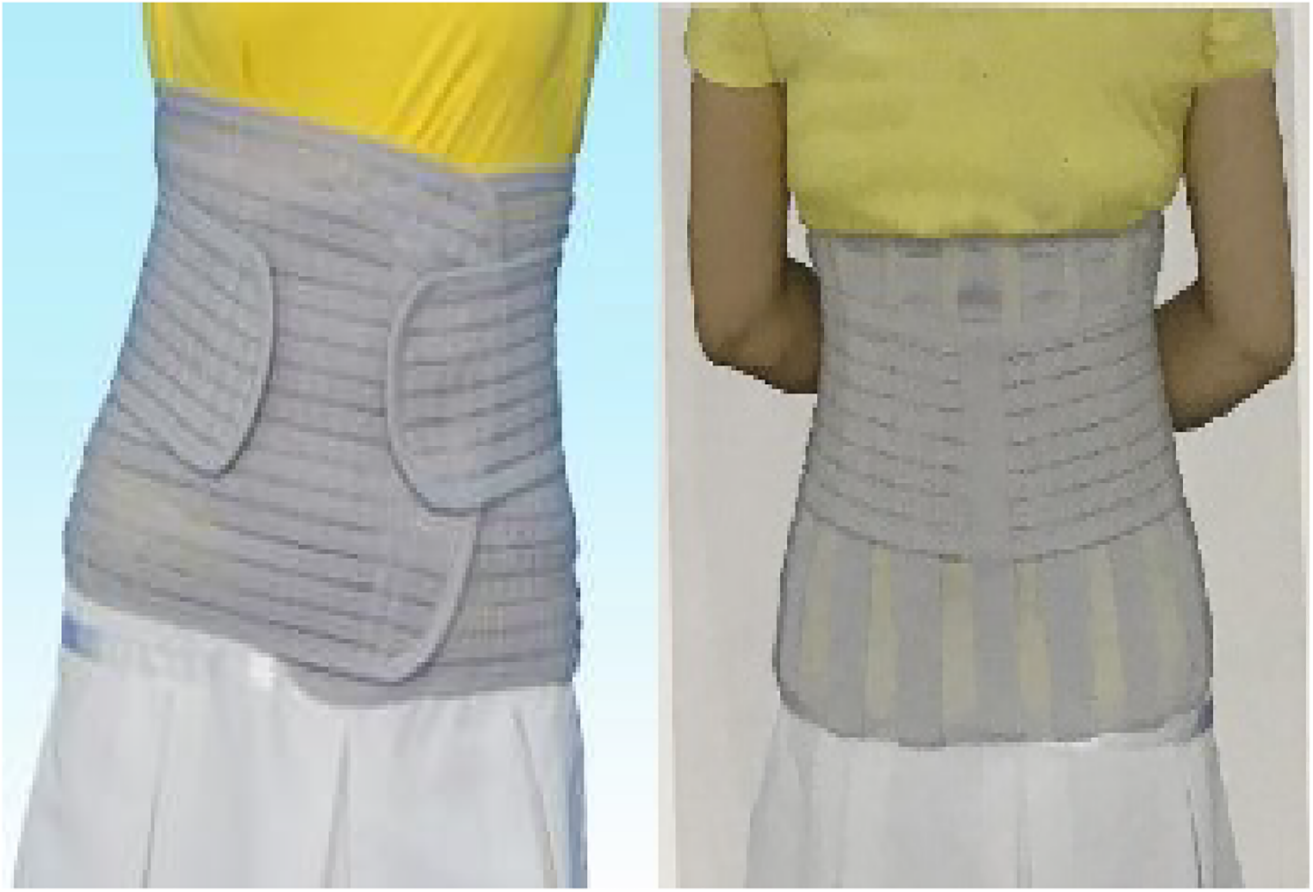
Soft brace.

Patients with a postoperative treatment and rehabilitation exercises after PVP and professionals in the brace room originally made braces by measuring the patients’ parameters. Among them, a rigid brace was a thoracolumbar frame orthosis composed of a subclavian-to-sternal stalk, with two transverse fingers on the navel to the pubic symphysis, and back from the injured vertebrae through a “three-point compression” system. A bracket is connected to the space in front of the front and rear pressure points (Figure 1). A soft brace consists of a circular waistline covering the thoracolumbar spine (Figure 2).

The improvement of pains in the event of refracture was measured based on the VAS score, the functional improvement of patients was measured based on the ODI score, and the overall recovery of patients was measured based on the Japanese Orthopaedic Association (JOA) score. The incidence of vertebral refracture was evaluated through positive and lateral x-ray films as well as MRI results during the follow-up period.

SPSS version 19.0 was used to analyze the data. Age, BMI, BMD, VAS, ODI, and JOA scores were compared between both groups through independent sample t-tests, and paired sample t-tests were performed for the intragroup comparison. A chi-square test was performed on sex and the incidence of refracture. *P* < 0.05 indicated a statistically significant difference.

## Results

### The baseline data comparison

Compared to the general data, all patients after PVP were followed up for 8–24 months (mean 22.4 months). The follow-up data were as follows (Tables 1–3). A chi-square test was used for counting data such as sex between the refracture and non-refracture group (Table 1). There were 29 male and 71 female patients in the non-brace-wearing group (age range 65–82 years; mean age 70.4 ± 3.8 years), whose preoperative BMD was in the range of −2.5 to 4.4 (mean −3.34 ± 0.3); while in the rigid brace group, there were 23 male and 77 female patients (age range 65–84 years; mean age 72.4 ± 3.7 years), whose preoperative BMD was in the range of −2.5 to 4.0 (mean −3.24 ± 0.3). The age range of patients in the soft brace group was 62–85 years (mean 71.4 ± 4.0 years), whose preoperative BMD was in the range of −2.5 and 4.0 (mean −3.16 ± 0.2). There was no significant difference among the three groups in age, sex, BMI, BMD, preoperative dysfunction index (ODI), or VAS of preoperative pains. In the measurement data, such as age, preoperative BMI, preoperative BMD, preoperative VAS score, preoperative ODI, and Cobb angle, there was no significant difference between the recurrent and non-recurrent fracture group (*p* > 0.05, *α* = 0.05). The preoperative data between the two groups were balanced and comparable.

**Table 1:** Comparison of general data between the refracture and the non-refracture group (*n*, *x* ± *s*).

	Refracture (*n* = 63)	No refracture (*n* = 237)	*p*
Age	71.2 ± 6.5	72.45 ± 5.36	0.457
Sex (male/female)	23/40	45/192	0.532
BMI (kg/m^2^)	17.3 ± 6.25	17.42 ± 6.28	0.073
BMD (T)	−3.36 ± 0.24	−3.23 ± 0.23	0.618
VAS score	7.2 ± 0.8	7.5 ± 1.0	0.765
ODI (%)	73.2 ± 4.7	72.9 ± 5.2	0.431
Cobb angle (°) pre-operation	20.55 ± 3.10	21.14 ± 2.91	0.403
3 days after the operations	9.37 ± 3.28	9.38 ± 3.95	0.980
Final follow-up	9.15 ± 3.12	9.29 ± 2.76	0.899

BMI, body mass index; BMD, bone mineral density; VAS, visual analog scale; ODI, Oswestry Disability Index.

The chi-squared test was used for sex, and t-test was used for measurement data (p > 0.05, α = 0.05).

**Table 2:** Refracture site [*n* (%)].

	Original surgical vertebra	Adjacent surgical vertebra	Non-adjacent surgical vertebra	Total
T10	0	3	2	5
T11	1	7	5	13
T12	1	10	9	20
L1	1	9	5	15
L2	0	5	1	6
L3	0	2	1	3
L4	0	1	0	1
	3 (4.67%)	37 (58.73%)	23 (36.51%)	63 (100%)

**Table 3:** Refracture in different treatment groups (*n*).

	Original surgical vertebra	Adjacent surgical vertebra	Non-adjacent surgical vertebra	Total
Soft brace	0	10	2	12
Hard brace	1	12	3	16
Non-brace	2	15	18	35
total	3	37	23	63

### Evaluations of the fracture incidence in the treated groups differed

The VAS score of the brace-wearing group was significantly higher than that of the non-braced group. The difference was statistically significant (*t* = −2.67, *p* < 0.05). The ODI of the brace-wearing group was lower than that of the non-brace-wearing group (*p* < 0.05). The JOA score of the brace-wearing group was significantly higher than that of the non-brace-wearing group, between which there was a significant difference (*t* = 2.56, *p* < 0.01). The incidence of vertebral refracture in the brace-wearing group was lower than that in the non-brace-wearing group, between which there was a significant difference (*p* < 0.05) (Table 4).

**Table 4:** Treatment evaluation and incidence of refracture among different treatment methods (*n*, *x* ± *s*).

	Brace group (*n* = 200)			Non-brace group (*n* = 100)			*t*	*p*
	Pre-operation	3 month after the operations	Final follow-up	Pre-operation	3 month after the operations	Final follow-up		
VAS score	7.82 ± 0.44	1.45 ± 0.56	1.10 ± 0.46	7.56 ± 0.78	2.09 ± 0.85	2.31 ± 0.72	−2.67^a^	0.04
ODI (%)	77.3 ± 4.8	12.1 ± 3.2	10.6 ± 2.3	75.4 ± 3.7	13.9 ± 3.6	11.6 ± 3.1	2.89^a^	0.03
JOA score	7.2 ± 1.1	23.2 ± 0.8	27.9 ± 1.7	7.8 ± 0.6	20.7 ± 1.2	24.8 ± 1.0	2.56^a^	0.02
Refracture rate (%)	28/172 (28%)	35/65 (35%)	—	0.03

VAS, visual analog scale; ODI, Oswestry Disability Index; JOA, Japanese Orthopaedic Association; t, T-test.

^a^ *p* < 0.05, α = 0.05.

### A rigid brace and a soft brace were worn after three months to evaluate refracture incidence

A comparison of treatment evaluation and the incidence of refracture between the rigid and soft brace group was conducted 3 months postoperatively, between which there was no significant difference in the VAS (*p* > 0.05, *α* = 0.05) or the ODI score (*p* > 0.05, *α* = 0.05). The JOA score of the soft brace group was significantly higher than that of the rigid brace group, between which there was a significant difference (*t* = 2.42, *p* < 0.05). There was no significant difference in the incidence of vertebral refracture between the two groups (*p* > 0.05, *α* = 0.05) (Table 5).

**Table 5:** Evaluation of treatment and incidence of refracture in the hard brace group and soft brace group 3 months after operation (*n*, *x* ± *s*).

	Soft brace group (*n* = 100)		Rigid brace group (*n* = 100)		*t*	*p*
	Pre-operation	3 month after the operations	Pre-operation	3 month after the operations		
VAS score	7.31 ± 0.26	1.16 ± 0.52	7.56 ± 0.78	1.18 ± 0.62	−1.65^b^	0.232
ODI (%)	75.9 ± 4.6	11.3 ± 3.3	75.4 ± 3.7	11.5 ± 3.1	−1.89^b^	0.241
JOA score	6.9 ± 1.3	24.2 ± 1.1	7.1 ± 0.8	21.9 ± 0.9	2.42^a^	0.004
Refracture rate (%)	10/90 (10%)	12/88 (12%)	—	0.357

VAS, visual analog scale; ODI, Oswestry Disability Index; JOA, Japanese Orthopaedic Association; t, T-test.

^a^ *p* > 0.05, α = 0.05.

^b^ *p* < 0.05.

### An assessment of subsequent fracture incidence in the last follow-up was conducted with a group of participants wearing rigid and soft braces

The VAS, ODI, and JOA scores in the last follow-up of the rigid brace group were compared to those of the soft brace group. There was no significant difference in the VAS (*p* > 0.05, *α* = 0.05) or ODI score (*p* > 0.05, *α* = 0.05) of the soft and rigid brace groups. The improvement in the JOA score of the soft brace group was higher than that of the rigid brace group, between which there was a significant difference (*t* = 2.17, *p* < 0.05). There was no significant difference in the incidence of vertebral refracture between the two groups (*p* > 0.05, *α* = 0.05) (Table 6).

**Table 6:** Evaluation of treatment and incidence of refracture in the hard and soft brace groups during the last follow-up (*n*, *x* ± *s*).

	Soft brace group (*n* = 100)	Hard brace group (*n* = 100)	*t*	*p*
VAS score	1.06 ± 0.48	1.15 ± 0.56	−1.24^a^	0.537
ODI (%)	10.3 ± 2.9	10.9 ± 3.2	−1.88^a^	0.286
JOA score	25.6 ± 1.2	24.2 ± 1.0	2.17^b^	0.003
Refracture rate (%)	12/89 (12%)	16/83 (16%)	—	0.239

VAS, visual analog scale; ODI, Oswestry Disability Index; JOA, Japanese Orthopaedic Association; t, T-test.

^a^ *p* > 0.05, α = 0.05.

*^a^*p* < 0.05.

## Typical cases

### Case 1

**Figure 3:**
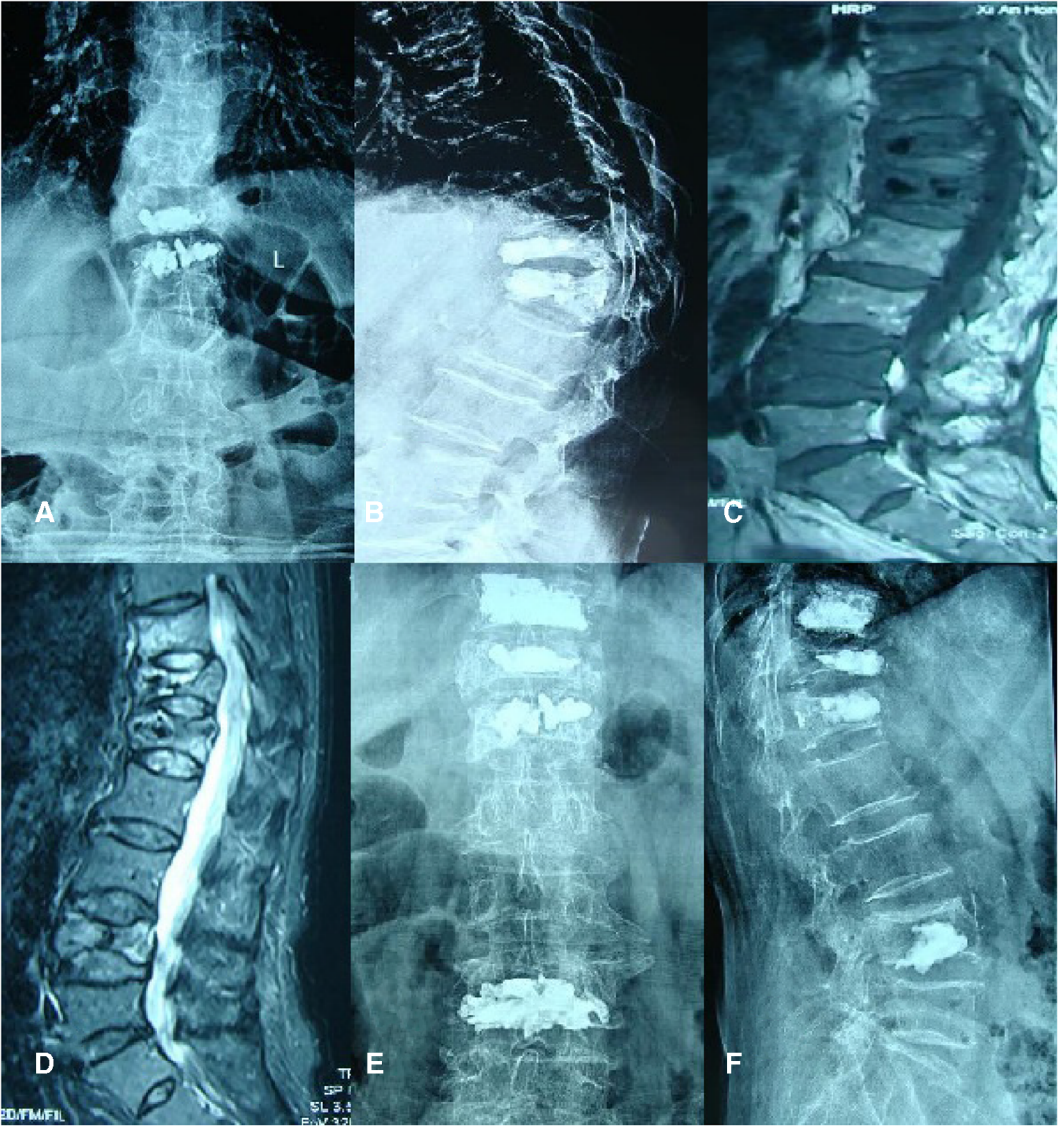
Case 1.

Case 1 was a 73-year-old woman who did not wear a brace after PVP, who felt low back pains 6 months after the operation. Anterior and lateral radiographs of the lumbar vertebrae were taken after the first PVP treatment. After admission, an MRI of the lumbar vertebrae showed fresh fractures at T11 and L4. Positive and lateral x-rays showed that the bone cement had diffused after the operation. In the last follow-up, the patient reported no pain or discomfort, and no new fracture was detected through imaging (Figure 3).

### Case 2

**Figure 4:**
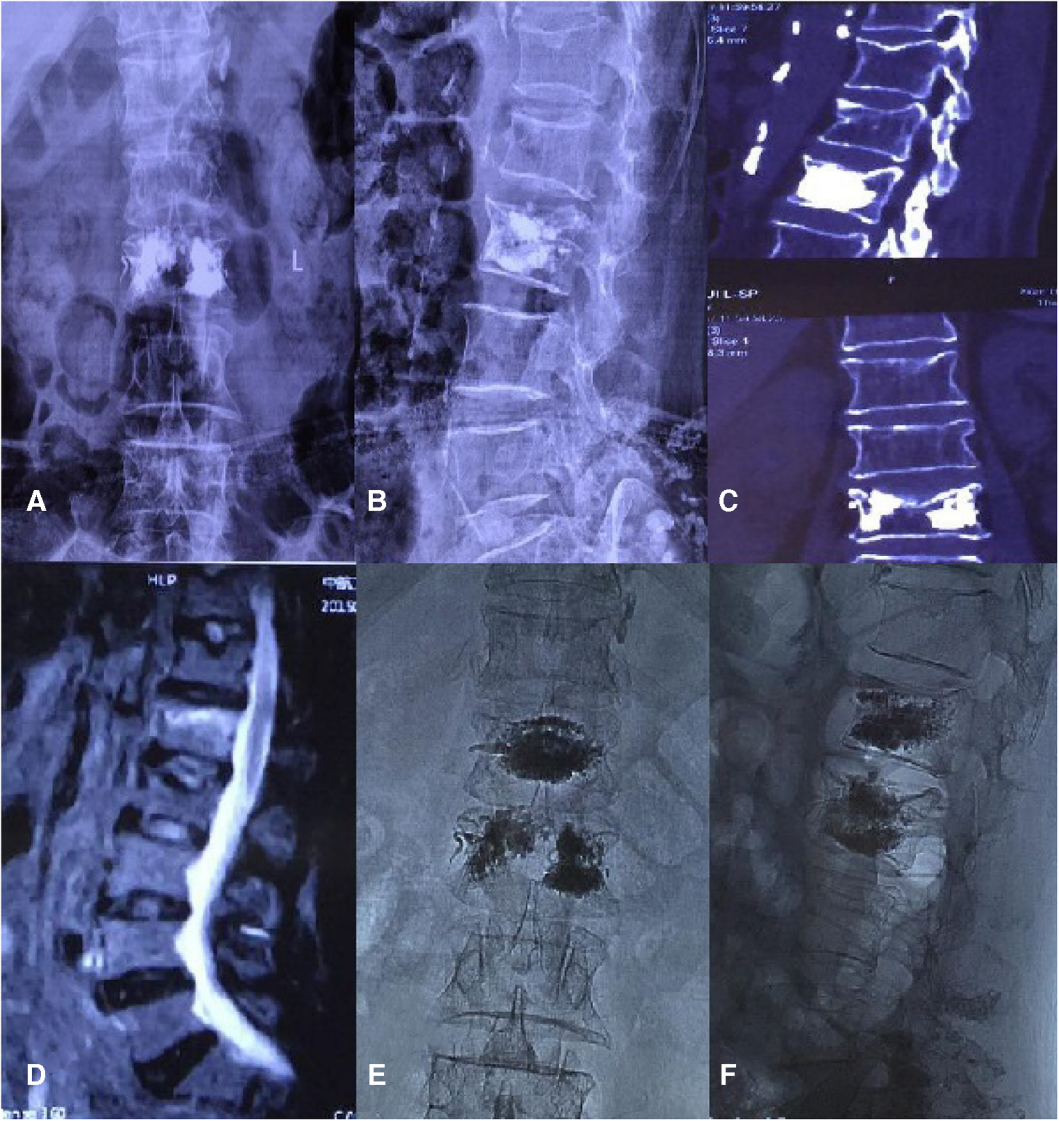
Case 2.

In case 2, a 75-year-old woman wore a soft brace after PVP but developed low back pains 19 months after the surgery. Anterior and lateral radiographs of the lumbar vertebrae were taken after the first PVP treatment. After admission, a CT scan of her spine failed to reveal any apparent fracture band, but a lumbar MRI examination showed a fresh fracture in L1. After an operation and the last follow-up, the x-ray showed that the bone cement was diffused, and in the last follow-up, the patient reported no low back pain (Figure 4).

### Case 3

**Figure 5:**
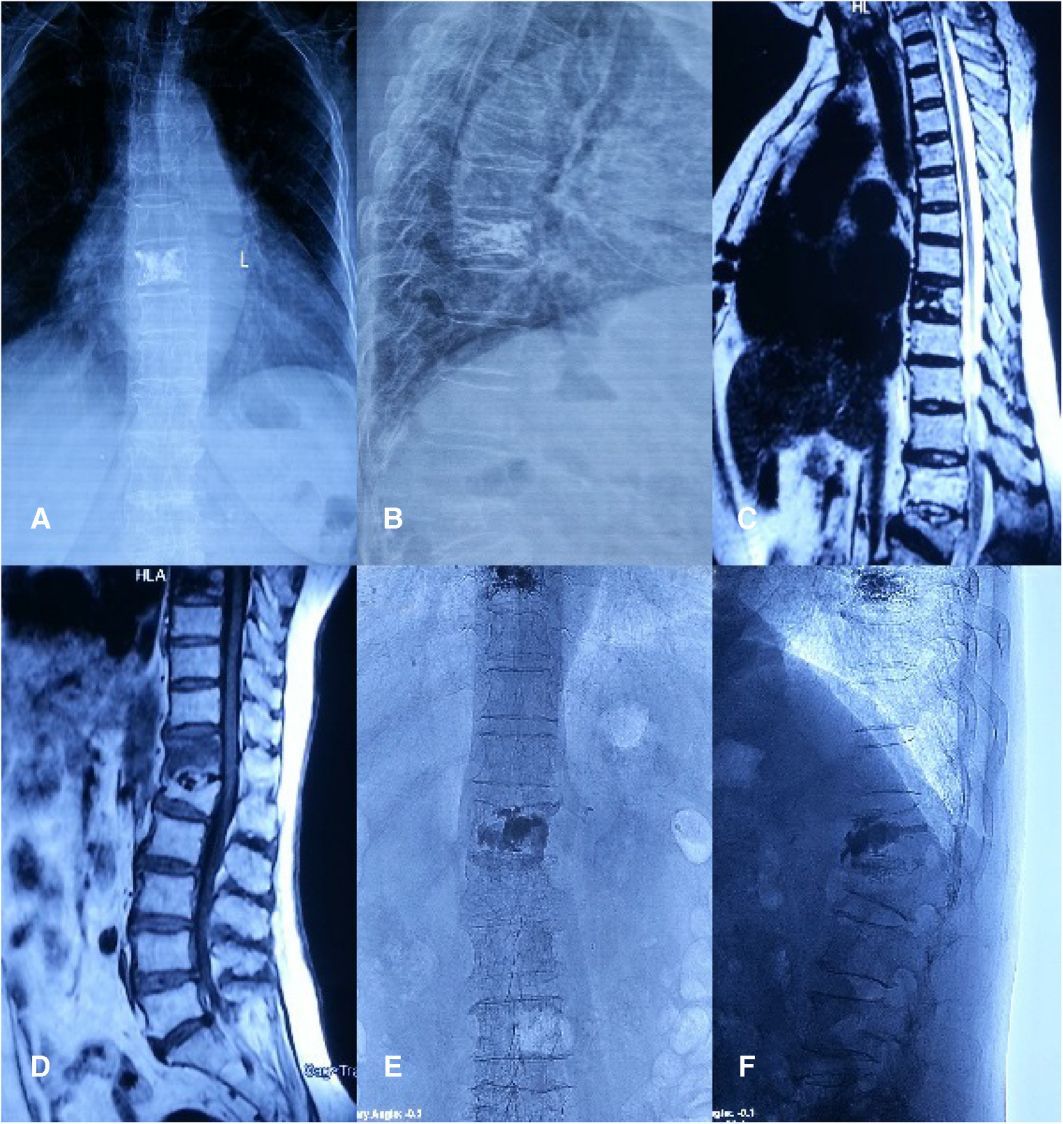
Case 3.

Case 3 was an 80-year-old woman with low back pain 1 year after PVP, who was wearing a rigid brace. After the first PVP treatment, the anterior and lateral radiographs of the lumbar vertebrae showed that the position of the bone cement was acceptable, and there were no vertebral displacements. After admission, an MRI of the lumbar vertebrae showed fresh fractures at the T12 vertebrae. A postoperative x-ray showed that the bone cement was filled. In the last follow-up, the patient had recovered well, there was no fresh fracture at the time of reexamination, the position of bone cement was not available, and the imaging results showed no obvious abnormalities after PVP (Figure 5).

## Discussion

The proportion of elderly people with osteoporosis is gradually increasing. Currently, there are about 200 million patients worldwide with osteoporosis, most of whom are in China. According to relevant experts, it is predicted that there will be 4.83 million patients with osteoporotic fractures in China in 2035 and 5.99 million in 2050 (8). Currently, vertebral compression fractures have become one of the most common complications among patients with osteoporosis, seriously affecting human health and quality of life. PVP is an extremely effective method for the clinical treatment of osteoporotic vertebral compression fractures, in which a rapid relief of patients’ symptoms and a shortened recovery time have been recognized by a wide range of experts and scholars. However, cases of refracture after PVP also occur frequently, which greatly impacts patients at the same time. Long-term stress distribution leads to the degeneration of adjacent vertebrae and increases the risk of vertebral refracture (9, 10). Tanigawa et al. (11) found that the incidence of postoperative vertebral fractures was 33.5% after a mean follow-up of 31 months among 194 patients with osteoporotic vertebral fractures undergoing PVP. Of the patients, 63.1% had clinical vertebral fractures and 36.9% had non-clinical vertebral fractures. At present, experts (12–26) at home and abroad have followed and reported on different cases of prevention, treatment, and regression of vertebral body refracture after vertebral compression fractures, but there are still a lot of controversies surrounding the treatment, follow-up time, sample size, and other factors after surgeries, together with the great difference in support for the prevention of refracture after vertebroplasty in different studies. In this study, we collected data from 1,167 patients who underwent vertebroplasty at our hospital between January 2017 and December 2018 and comprehensively analyzed the effect of bracing on refractures after a vertebroplasty. The analysis showed that the irregular wearing of braces was a risk factor for secondary fractures after a vertebroplasty for osteoporotic vertebral compression fractures, and that soft braces were more advantageous for the improvement of lumbar spine functions of patients.

Stenting, as a routine treatment for osteoporotic compression fractures, is still commonly used in clinical practice despite the lack of high-quality evidence. In the present study, contrasting outcomes were demonstrated between patients treated with and without bracing, with equal and significant improvements in pain, functional activities, and disabilities, especially for patients with braces. In a study by Kim et al. (27), significant improvements in pain and disability were observed in all three groups.

There have been some reports (27–35) examining the supporting effect of soft and hard braces. However, the concept and execution of support are essentially different for soft and hard braces. A soft brace support is quite gentle, and has been designed to allow more freedom for movement. As a patient moves backward in a convexity, he or she is prompted to straighten the back by relaxing the extensor muscles. The reported data suggest that it will lead to improved posture and muscle strength over time. One study reported (33) that no significant differences were found in pain (VAS scores) or disability (Oswestry Low Back Pain Disability Questionnaire scores) after 1 month. However, after the bracing sessions, there were better results after 3 and 6 months. A reasonable conclusion is that through biofeedback, patients are trained to maintain better posture through soft bracing, which is a benefit that is more durable than rigid external fixation alone. During the follow-up in the present study, it was also found that a soft support was more advantageous for the functional improvement of the low back.

During our bracing for postoperative refractures of osteoporotic vertebral compression fractures, patients treated with soft bracing had better pain control and respiratory function than those treated with rigid bracing after 1 and 3 months, and also showed a greater improvement in quality of life. In terms of radiological outcomes, soft bracing was at least as effective as hard bracing in stabilizing osteoporotic spine fractures. In the present study, soft bracing proved to be safe and effective in the treatment for postoperative refractures of osteoporotic vertebral compression fractures, with better functional outcomes and fewer complications than previous standard hard braces.

There are still some shortcomings in this study. The main results are as follows: (1) patients with comparable general conditions were selected as much as possible, but a retrospective analysis was inevitable as it would lead to biases; (2) the study was a single-center study, where the data could not represent the majority of patients, so a multicenter study was needed to examine a wider population; and (3) in the future, a larger sample size and longer-term follow-up will be needed, and a strict prospective study needs to be designed to provide more scientific as well as rigorous research results.

## Conclusion

Even though PVP is an effective method for treating OVCFs, it leads to long-term complications. After a PVP procedure, patients are advised to wear their braces conscientiously, which enables them to avoid a series of related complications as soon as possible meanwhile significantly reducing the risk of vertebral refracture. Not only does this improve the quality of life of patients, but the burden on their families and society is also reduced. Bracing improves the prognosis in terms of quality of life and postoperative subjective perception. The presence of a brace can relieve postoperative residual pains. In contrast, wearing a soft brace can give patients a better medical experience.

## Data Availability

The raw data supporting the conclusions of this article will be made available by the authors, without undue reservation.
